# Drebrin Isoforms Critically Regulate NMDAR- and mGluR-Dependent LTD Induction

**DOI:** 10.3389/fncel.2018.00330

**Published:** 2018-10-08

**Authors:** Hiroki Yasuda, Nobuhiko Kojima, Kenji Hanamura, Hiroyuki Yamazaki, Kenji Sakimura, Tomoaki Shirao

**Affiliations:** ^1^Department of Neurobiology and Behavior, Graduate School of Medicine, Gunma University, Maebashi, Japan; ^2^Education and Research Support Center, Graduate School of Medicine, Gunma University, Maebashi, Japan; ^3^Division of Physiology, Faculty of Medicine, Saga University, Saga, Japan; ^4^Faculty of Life Sciences, Toyo University, Itakura, Japan; ^5^Department of Cellular Neurobiology, Brain Research Institute, Niigata University, Niigata, Japan

**Keywords:** drebrin isoforms, NMDAR-dependent LTD, mGluR-dependent LTD, knockout mouse, development

## Abstract

Drebrin is an actin-binding protein that is preferentially expressed in the brain. It is highly localized in dendritic spines and regulates spine shapes. The embryonic-type (drebrin E) is expressed in the embryonic and early postnatal brain and is replaced by the adult-type (drebrin A) during development. In parallel, NMDA receptor (NMDAR)-dependent long-term depression (LTD) of synaptic transmission, induced by low-frequency stimulation (LFS), is dominant in the immature brain and decreases during development. Here, we report that drebrin regulates NMDAR-dependent and group 1 metabotropic glutamate receptor (mGluR)-dependent LTD induction in the hippocampus. While LFS induced NMDAR-dependent LTD in the developing hippocampus in wild-type (WT) mice, it did not induce LTD in developing drebrin E and A double knockout (DXKO) mice, indicating that drebrin is required for NMDAR-dependent LTD. On the other hand, LFS induced robust LTD dependent on mGluR5, one of group 1 mGluRs, in both developing and adult brains of drebrin A knockout (DAKO) mice, in which drebrin E is expressed throughout development and adulthood. Agonist-induced mGluR-dependent LTD was normal in WT and DXKO mice; however, it was enhanced in DAKO mice. Also, mGluR1, another group 1 mGluR, was involved in agonist-induced mGluR-dependent LTD in DAKO mice. These data suggest that abnormal drebrin E expression in adults promotes group 1 mGluR-dependent LTD induction. Therefore, while drebrin expression is critical for NMDAR-dependent LTD induction, developmental conversion from drebrin E to drebrin A prevents robust group 1 mGluR-dependent LTD.

## Introduction

Synaptic plasticity is the change in efficacy of synaptic transmission induced by activity, which is hypothesized to be a molecular mechanism of learning and memory. Actin filaments (F-actin) form major cytoskeletons in dendritic spines (Matus, 2005) and remodeling of F-actin underlies structural plasticity, with alteration of postsynaptic spine morphology being associated with synaptic plasticity (Matsuzaki et al., 2004; Okamoto et al., 2004; Zhou et al., 2004; Honkura et al., 2008; Shirao and González-Billault, 2013; Caroni et al., 2014). Drebrin is an F-actin-binding protein that modulates actin remodeling by elongating the helical crossover of F-actin (Sharma et al., 2012; Grintsevich, 2017). Drebrin is classified into two major alternatively spliced isoforms, embryonic-type and adult-type (drebrin E and drebrin A, respectively; Shirao and Sekino, 2017). Drebrin A is neuron-specific, appears at nascent postsynaptic sites, and forms an F-actin platform for the molecular assembly of postsynaptic proteins, such as PSD-95, spikar and glutamate receptors (Takahashi et al., 2003; Aoki et al., 2005; Yamazaki et al., 2014). Additionally, we have recently shown that long-term potentiation (LTP) and context-dependent fear memory are impaired in adult drebrin A knockout (DAKO) mice (Kojima et al., 2010, 2016), indicating that drebrin plays a pivotal role in synaptic plasticity (Sekino et al., 2017).

Drebrins E and A have similar primary amino acid sequences except that drebrin A has an insertion of 46 amino acids (Kojima et al., 1993) in the middle of the molecule named Ins2 (Kojima, 2017). Drebrin has an actin depolymerizing factor homology domain, two actin-binding regions (Hayashi et al., 1999; Xu and Stamnes, 2006; Grintsevich et al., 2010), and two Homer-binding sequences (Shiraishi-Yamaguchi et al., 2009; Yamazaki and Shirao, 2017). However, the time courses of drebrin E and A expression in the brain are quite different from each other. Drebrin E is expressed in the embryonic and early postnatal brain, is involved in neuronal migration and axonal growth (Hanamura, 2017), and starts to be replaced with drebrin A at around postnatal day 8 (P8; Aoki et al., 2005), when dendritic spine numbers are rapidly increasing (Yuste and Bonhoeffer, 2004). The timing of drebrin A appearance is relevant for its regulatory role in spine formation. On the other hand, physiological consequences of drebrin E disappearance in the adult brain are not clear.

Long-term depression (LTD), an activity-dependent decrease in synaptic efficacy, is induced by NMDA receptors (NMDARs) or group 1 metabotropic glutamate receptors (mGluRs), and is expressed by reduced numbers of AMPA receptors (AMPARs) at synapses in the hippocampus (Malenka and Bear, 2004; Huganir and Nicoll, 2013). NMDAR-dependent LTD is hypothesized to underlie memory flexibility, in which stored memories are renewed by new information (Caroni et al., 2014; Connor and Wang, 2016). On the other hand, mGluR-dependent LTD is enhanced by stress (Chaouloff et al., 2007) and is increased in brain disorders including autism, and is supposed to cause memory deficits (Lüscher and Huber, 2010; Niswender and Conn, 2010; Bhakar et al., 2012). Also, NMDAR-dependent LTD is dominant in the developing brain and is decreased in the adult brain (Dudek and Bear, 1993; Kemp et al., 2000). The coincidence of drebrin E and dominant LTD induction suggests that drebrin E is involved in LTD. In the present study, we examined LTD induction in the CA1 region of the hippocampus in developing and adult DAKO and drebrin E and A double knockout (DXKO) mice (Kajita et al., 2017), and investigated the roles of drebrin isoforms in LTD induction. Here we discuss the differential role of drebrin isoforms in NMDAR-dependent LTD. We also report abnormal induction of group 1 mGluR-dependent LTD in the developing and adult hippocampus in DAKO mice.

## Materials and Methods

Animal use and all experimental procedures were approved by the Ethical Committee for Animal Experiments of Gunma University (#50095, 07-114, 09-022, 12-032, 14-030) and by the institutional review committees at Niigata University (year 2007, #41), and all experiments were performed in accordance with the guidelines of these committees.

### Slice Electrophysiology

Synaptic transmission was recorded from mouse hippocampal slices as described previously (Yasuda et al., 2003; Yasuda and Mukai, 2015; Kojima et al., 2016). Briefly, slices were cut from septal hippocampi of DAKO and DXKO mice or their WT littermates in ice-cold oxygenated (95% O_2_/5% CO_2_) artificial cerebrospinal fluid (ACSF) containing (in mM) 119 NaCl, 2.5 KCl, 26.2 NaHCO_3_, 1 NaH_2_PO_4_, 4 CaCl_2_, 4 MgSO_4_ and 11 glucose (pH 7.4) and incubated for at least 2 h. Two slices were placed in a submersion-type recording chamber mounted on an upright microscope (BX51WI, Olympus, Tokyo, Japan) and perfused with the same oxygenated ACSF containing 100 μM picrotoxin at 30°C. Field excitatory postsynaptic potentials (fEPSPs) with an amplitude of approximately 0.3 mV were evoked with a stimulating glass electrode containing the same ACSF placed in the stratum radiatum and recorded in the CA1 region using a Multiclamp 700B amplifier (Molecular Devices, Sunnyvale, USA). Acquisition and measurement of fEPSP slopes were performed using custom Igor Pro (WaveMetrics, Lake Oswego, OR, USA) software routines. Basal synaptic transmissions were obtained at 0.05 Hz, and LTD was induced by applying 1 Hz 15 min stimulation or RS-DHPG (DHPG).

### Drugs

D-APV, MPEP, YM 298198 and DHPG were from Tocris Bioscience (Bristol, UK). Other chemicals were from Wako Pure Chemical Industries (Osaka, Japan).

### Protein Sample Preparation and Western Blotting

Mice were sacrificed by cervical dislocation, and removed brains were washed in ice-cold phosphate buffered saline. The synaptosomal fraction was prepared in sucrose density gradients essentially as described by Gray and Whittaker (1962). Briefly, the cerebral cortices were homogenized in a glass-Teflon homogenizer in nine volumes of 0.32 M sucrose, 1 mM NaHCO_3_, 1 mM MgCl_2_, 0.5 mM CaCl_2_ containing protease inhibitors (Complete™; Roche, Basel, Switzerland), and centrifuged at 1,400× *g* for 10 min at 4°C. The supernatant was recentrifuged at 13,800× *g* for 20 min at 4°C to give a pellet. After resuspending the pellet in 0.32 M sucrose, 1 mM NaHCO_3_, this crude synaptosomal fraction was layered on top of a three-layered discontinuous sucrose density gradient (0.8, 1.0 and 1.2 M sucrose layers), and centrifuged at 82,500× *g* for 2 h at 4°C. A needle and syringe was then used to collect the synaptosomal fraction from the interface between 1.0 M and 1.2 M sucrose layers. The synaptosomal fraction was diluted in 0.32 M sucrose, 1 mM NaHCO_3_. For extraction experiments, the synaptosomal fraction was homogenized in a Teflon homogenizer in a buffer solution containing 1% Triton X-100 or 1 M NaCl, and centrifuged at 165,000× *g* to separate the cytosolic fraction from the membrane fraction. Protein concentration of samples was determined using a DC protein assay kit (Bio-Rad Laboratories, Hercules, CA, USA). The samples were denatured in sodium dodecyl sulfate (SDS) sample buffer. Equal amounts of protein were separated by SDS-polyacrylamide gel electrophoresis, transferred onto polyvinylidene difluoride membranes (Merck Millipore, Darmstadt, Germany), and probed with the primary antibodies, anti-drebrin (clone M2F6; MBL, Nagoya, Japan; Shirao and Obata, 1986), anti-β-tubulin (clone 152H6; Kojima et al., 1988) and anti-β-actin (AC-15; Sigma, St. Louis, MO, USA). After incubation with horseradish peroxidase-conjugated second antibody, blots were developed using an enhanced chemiluminescence system (ECL; GE Healthcare, Piscataway, NJ, USA). Immunoreactive signals were visualized using an image analyzer (LAS-3000; Fujifilm, Tokyo, Japan) and quantified with the public domain software, ImageJ (available at: http://rsb.info.nih.gov/ij/).

### Statistical Analyses

Results are reported as the mean ± SEM. The normality of distribution of each dataset was checked by Shapiro-Wilk and Kolmogorov-Smirnov tests using EZR (Easy R) software (Kanda, 2013). The statistical significance of differences between two groups was analyzed using Student’s *t* test in Excel. For multiple comparisons, one-way ANOVA with the Tukey–Kramer test was performed using EZR.

## Results

### NMDAR-Dependent LTD is Not Induced in the Hippocampus of DXKO Mice

**Figure 1:**
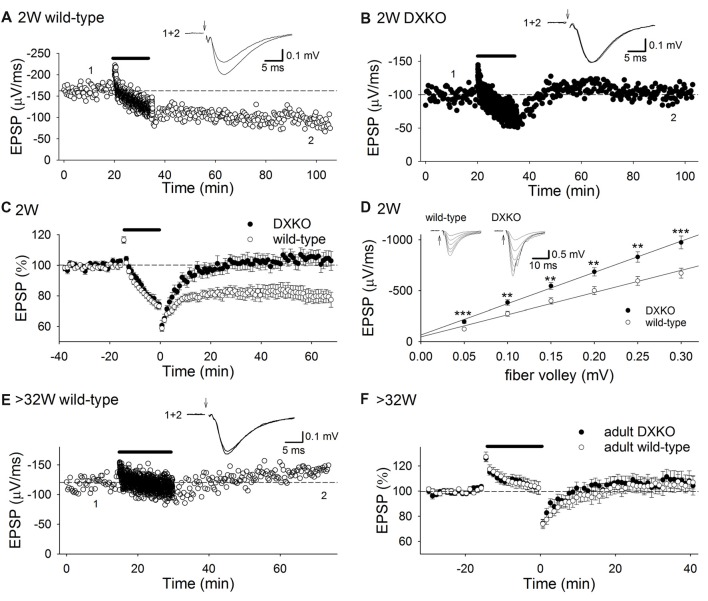
Low-frequency stimulation (LFS) does not induce long-term depression (LTD) in DXKO mice. **(A)** Example of LTD in a P20 wild-type (WT) mouse. **(B)** Time course of field excitatory postsynaptic potential (fEPSP) slopes before, during and after LFS in a P18 DXKO mouse. LFS did not induce LTD. **(C)** Average time course of LTD experiments in P18–20 WT (*n* = 14 from four mice) and DXKO mice (*n* = 12 from five mice). NMDA receptor (NMDAR)-dependent LTD was not induced in P18–20 DXKO mice, suggesting that either drebrin E or A is required in NMDAR-dependent LTD induction at these ages. **(D)** Input-output relationship in P18–20 WT (*n* = 17 from four mice) and DXKO mice (*n* = 21 from four mice; **p < 0.01; ***p < 0.001; Student’s *t*-test). **(E)** Example of the effects of LFS on fEPSPs in a P257 (postnatal 36W) WT mouse. **(F)** Averaged time course of fEPSP slopes before, during, and after LFS in >32W WT (*n* = 15 from four mice) and DXKO mice (*n* = 12 from four mice). LFS did not induce LTD in DXKO mice older than 32 weeks.

Initially, we tested whether 1 Hz 15 min low-frequency stimulation (LFS) induces NMDAR-dependent LTD in developing DXKO mice in which neither drebrin E nor A accumulates in dendritic spines (Kajita et al., 2017). We found that LFS did not induce LTD in P18–20 DXKO mice, but it did induce LTD in WT mice (Figures 1A–C; WT, 80.2 ± 4.5% of baseline 60 min after LFS, *n* = 14 from four mice; DXKO, 105.1 ± 4.1%, *n* = 12 from five mice; *p* < 0.0005, Student’s *t*-test). LTD induced by LFS is NMDAR-dependent in WT mice (Mulkey and Malenka, 1992; Dudek and Bear, 1993; Kemp et al., 2000; Malenka and Bear, 2004), suggesting that drebrin is necessary for the induction of NMDAR-dependent LTD in the P18–20 hippocampus. In addition, input-output relationship in DXKO mice was significantly elevated compared to WT (Figure 1D; WT, *n* = 17 from four mice; DXKO, *n* = 21 from four mice). Removal of AMPARs from postsynaptic sites through endocytosis underlies NMDAR-dependent LTD (Malenka and Bear, 2004; Huganir and Nicoll, 2013). Therefore, lack of LTD remains AMPARs at postsynaptic sites without removal, induces elevation of input-output relationship, and may cause higher excitability. Elevation of input-output relationship is not caused by enhancement of LTP because the amplitude of LTP was not different between WT and DXKO mice (Supplementary Figure S1A; WT, *n* = 11 from four mice; DXKO, *n* = 9 from four mice). Paired-pulse ratio (PPR; Supplementary Figure S1B; WT, *n* = 10 from three mice; DXKO, *n* = 12 from three mice) and posttetanic potentiation (PTP; Supplementary Figure S1C; WT, *n* = 9 from three mice; DXKO, *n* = 10 from three mice) were not different between WT and DXKO mice, suggesting that presynaptic release properties are not changed in DXKO mice. Therefore, lack of NMDAR-dependent LTD most likely causes elevation of input-output relationship in developing DXKO mice.

We also examined LTD in adult DXKO mice, because LTP is impaired in adult DAKO mice (Kojima et al., 2016). LFS induced no LTD both in adult WT (Figures 1E,F; 104.6 ± 4.7% of baseline 40 min after LFS, *n* = 15 from four mice) and DXKO mice (110.1 ± 6.5%, *n* = 12 from four mice).

### mGluR5-Dependent LTD Is Enhanced in the Hippocampus of Developing DAKO Mice

**Figure 2:**
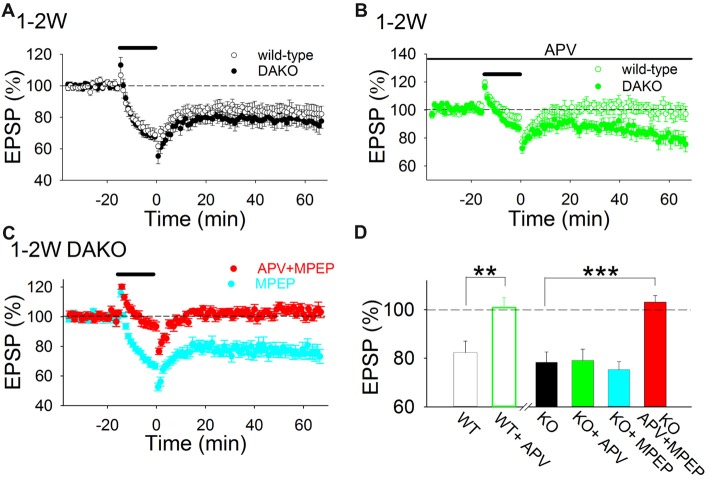
LTD Is both NMDAR- and metabotropic glutamate receptor (mGluR)5-dependent in the developing hippocampus of drebrin a knockout (DAKO) mice. **(A)** Average time course of LTD in 1–2-week postnatal WT (*n* = 13 from four mice) and DAKO mice (*n* = 14 from four mice). **(B)** Average time course of LTD in the absence or presence of D-APV in 1–2-week-old WT (*n* = 9 from four mice) and DAKO mice (*n* = 12 from five mice). D-APV completely blocked LTD in developing WT mice, however, D-APV did not affect LTD in developing DAKO mice. **(C)** Average time course of LTD in the presence of MPEP (*n* = 10 from four mice) or D-APV plus MPEP (*n* = 13 from seven mice) in 1–2-week-old DAKO mice. D-APV and MPEP were applied throughout experiments. LTD was not blocked by MPEP treatments; however, LTD was inhibited when D-APV and MPEP were applied simultaneously in developing DAKO mice. **(D)** Summary of the effects of D-APV and MPEP on LTD in 1–2-week-old WT and DAKO mice. The percentage of fEPSPs 55–60 min after LFS is shown. **p < 0.01, Student’s *t*-test; ***p < 0.001, one-way ANOVA with Tukey–Kramer test.

To investigate whether LTD is also altered in DAKO mice, we first examined whether the amplitude of LTD induced by LFS in developing DAKO mice was different from LTD in age-matched WT littermates. LFS induced LTD equally in 1–2 week postnatal WT and DAKO mice (Figure 2A; WT, 82.4 ± 4.7%, *n* = 13 from four mice; DAKO, 78.3 ± 4.3%, *n* = 14 from four mice). The input–output relationship was not different between developing WT and DAKO mice (Supplementary Figure S2; control, *n* = 11 from four mice; KO, *n* = 13 from four mice).

D-APV (50 μM), an NMDAR antagonist, completely inhibited LTD in 1–2-week-old WT mice (Figures 2B,D; D-APV, 100.9 ± 4.2%, *n* = 9 from four mice; significantly different from control at *p* < 0.01, Student’s *t*-test). This is consistent with previous studies showing that single pulse LFS induces NMDAR-dependent LTD in the CA1 region of the immature hippocampus (Mulkey and Malenka, 1992; Dudek and Bear, 1993; Kemp et al., 2000; Malenka and Bear, 2004). However, D-APV did not significantly block LTD in DAKO mice (Figures 2B,D; D-APV, 79.1 ± 4.6%, *n* = 12 from five mice).

Because mGluR5, a group 1 mGluR, also mediates LTD in the hippocampus (Bear et al., 2004; Malenka and Bear, 2004; Lüscher and Huber, 2010; Yasuda and Mukai, 2015), we tested whether simultaneous application of an mGluR5 antagonist, MPEP (10 μM) and D-APV inhibits LTD in developing DAKO mice. Simultaneous application of MPEP and D-APV blocked LTD in DAKO mice (Figures 2C,D; D-APV plus MPEP, 103.1 ± 2.7%, *n* = 13 from seven mice, which was significantly different from control at *p* < 0.0005, one-way ANOVA with Tukey–Kramer test), although MPEP did not block LTD (Figures 2C,D; MPEP, 75.3 ± 3.3%, *n* = 10 from four mice). This indicates that similar to LTD induced by 5 Hz stimulation in developing rat hippocampus (Oliet et al., 1997; Yasuda and Mukai, 2015), LTD induced by 1 Hz LFS in developing DAKO mice in the present study can be mediated by either NMDARs or mGluRs. However, NMDAR- and mGluR-dependent LTD are not additively induced; the amplitude of LTD in control conditions was similar to those of NMDAR-dependent LTD in the presence of MPEP and mGluR-dependent LTD in the presence of D-APV in DAKO mice (Figure 2D). These LTD might partially inhibit to induce each other in control conditions as reported in the basal amygdala (Clem and Huganir, 2013) and the amplitude of LTD was not the sum of those of both LTD.

**Figure 3:**
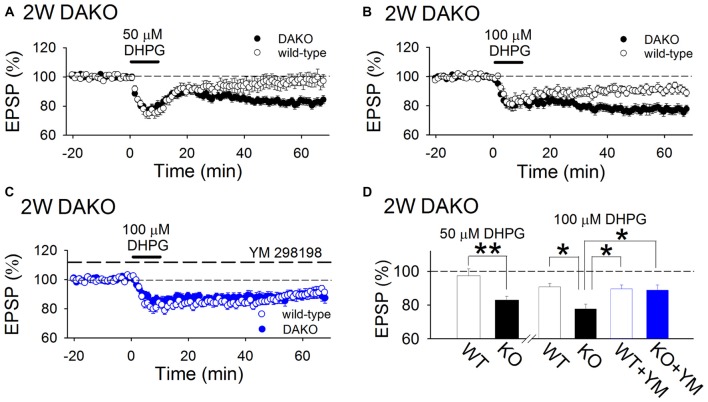
mGluR-dependent LTD Is enhanced in developing DAKO mice. **(A)** Average time course of the effects of 50 μM DHPG on synaptic transmission in the 2-week-old hippocampus of WT (*n* = 14 from five mice) and DAKO mice (*n* = 18 from four mice). A low concentration of DHPG induced LTD in DAKO mice, although it did not exert long-term effects on synaptic transmission in WT mice. **(B)** Average time course of LTD induced by 100 μM DHPG in the 2-week-old hippocampus of WT (*n* = 11 from five mice) and DAKO mice (*n* = 19 from five mice). **(C)** Average time course of 100 μM DHPG-induced LTD in the presence of 5 μM YM 298198 in WT (*n* = 16 from four mice) and DAKO mice (*n* = 18 from five mice). **(D)** Summary of DHPG-induced LTD experiments in developing WT and DAKO mice (**p < 0.01; Student’s *t*-test; *p < 0.05; one-way ANOVA with Tukey–Kramer test).

Next, we examined whether agonist-induced mGluR-dependent LTD is affected in developing DAKO mice. A low concentration (50 μM) of DHPG, a group 1 mGluR agonist, did not show significant long-term effects in WT mice; however, it induced LTD in DAKO mice (Figures 3A,D; WT, 97.4 ± 4.1%, *n* = 14 from five mice; DAKO, 82.8 ± 2.5%, *n* = 18 from four mice; *p* < 0.01, Student’s *t*-test). Although 100 μM DHPG induced LTD in WT mice (Figures 3B,D; 90.9 ± 2.0%, *n* = 11 from five mice), LTD in DAKO mice was more robust (Figures 3B,D; 77.7 ± 2.8%, *n* = 19 from five mice; *p* < 0.05; one-way ANOVA with Tukey–Kramer test). DHPG activates both mGluR1 and mGluR5, and mGluR1 is also involved in DHPG-induced LTD (Volk et al., 2006; Kumar and Foster, 2007). Therefore, we tested the effects of an mGluR1 selective inhibitor, YM 298198, on DHPG-induced LTD. 5 μM YM 298198 did not affect 100 μM DHPG-induced LTD in WT mice (Figures 3C,D; 89.6 ± 2.4%, *n* = 16 from four mice). However, YM 298198 reduced DHPG-induced LTD in DAKO mice (Figures 3C,D; 88.8 ± 3.2%, *n* = 18 from five mice; significantly different from control DAKO mice at *p* < 0.05; one-way ANOVA with Tukey–Kramer test). These results indicate that group 1 mGluR-dependent LTD is enhanced in developing DAKO mice.

### mGluR5-Dependent LTD Is Induced in the Hippocampus of Adult DAKO Mice

**Figure 4:**
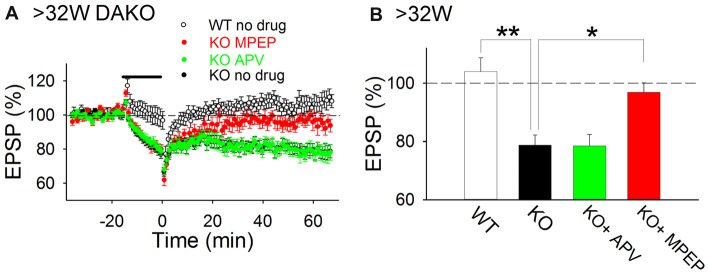
mGluR5-dependent LTD is aberrantly induced in adult DAKO mice. **(A,B)** Average time course **(A)** and summary **(B)** of LTD experiments in WT (*n* = 12 from four mice) and DAKO mice (control, *n* = 21 from eight mice; D-APV, *n* = 12 from three mice; MPEP, *n* = 16 from eight mice) at >32 weeks postnatal (*p < 0.05; **p < 0.01; one-way ANOVA with Tukey–Kramer test).

Previously, we reported that LTP was impaired in adult DAKO mice (Kojima et al., 2016); therefore, we considered that other synaptic plasticity mechanisms might be abnormally induced in the hippocampus of adult DAKO mice. We tested whether LTD was induced in 33–53-week-old DAKO mice. LFS did not induce LTD in adult WT mice and occasionally caused a small enhancement of fEPSPs (Figure 4; 104.0 ± 4.7%, *n* = 12 from four mice). This result is consistent with previous studies showing that LTD is barely induced by single pulse LFS in the adult hippocampus (Dudek and Bear, 1993; Kemp et al., 2000). On the other hand, LFS induced LTD in adult DAKO mice (Figure 4; 78.7 ± 3.5%, *n* = 21 from eight mice; which was significantly different from the WT at *p* < 0.005, Tukey–Kramer test). LTD in adult DAKO mice was not associated with significant changes in paired-pulse ratio (Supplementary Figure S3; baseline, 1.38 ± 0.04; 60 min after LFS, 1.41 ± 0.04, *n* = 5 from two mice), suggesting that decreased transmitter release is unlikely to be involved. D-APV did not affect LTD (Figure 4; 78.6 ± 3.8%, *n* = 12 from three mice), but MPEP inhibited LTD in adult DAKO mice (Figure 4; MPEP, 96.9 ± 3.3%, *n* = 16 from eight mice, which was significantly different from control DAKO mice at *p* < 0.05, one-way ANOVA with Tukey–Kramer test). These results indicate that LTD induction in the hippocampus of adult DAKO mice is mediated by mGluR5s.

### Abnormal Accumulation of Membrane-Bound Drebrin in Synaptosomes in Adult DAKO Mice

**Figure 5:**
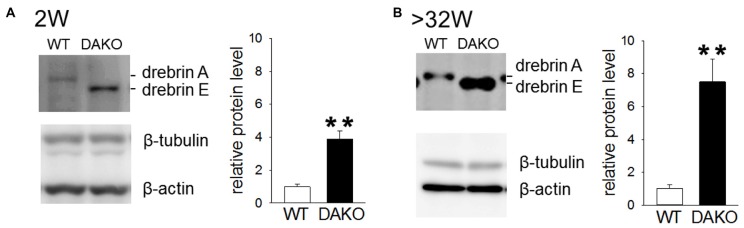
Drebrin E accumulates in synaptosome from DAKO mice. **(A)** Quantitative analyses of drebrin content in synaptosome from the cerebral cortices of developing WT (*n* = 3) and DAKO mice (*n* = 3). Equal amounts of protein (25 μg/lane) were subjected to SDS-PAGE/Western blot analysis. Blots were probed with antibodies against drebrin, β-tubulin and β-actin, and positive bands were visualized with enhanced chemiluminescence (ECL) system. The intensities of ECL signals for drebrin was quantified and the drebrin levels were shown as the ratio of DAKO to WT and are presented as means ± SEM. Note that the amount of drebrin in synpatosome of DAKO mice was significantlly higher thatn that of WT mice both in developing and adult mice (**p < 0.01; Student’s *t*-test,). **(B)** Quantitative analyses of drebrin content in synaptosome from adult WT (*n* = 4) and DAKO mice (*n* = 4). For original western blot images, see Supplementary Figure S4.

Previously, we reported that drebrin E is highly accumulated in synaptosomes prepared from DAKO mice, although the synaptosomal distribution of other synapse resident proteins, including CaMKIIα, Homer, PSD-95 and synaptophysin, is not different between WT and DAKO mice (Kojima et al., 2010). We confirmed that drebrin E was significantly more abundant in developing DAKO mice compared than drebrin A in WT mice (Figure 5A; WT, 1.00 ± 0.14, *n* = 3; DAKO, 3.88 ± 0.49, *n* = 3; *p* < 0.01, Student’s *t*-test). Also, drebrin E was still much more accumulated in synaptosomes from adult DAKO mice than drebin A in WT mice (Figure 5B; WT, 1.00 ± 0.25, *n* = 4; DAKO, 7.50 ± 1.40, *n* = 4; *p* < 0.01).

### mGluR-Dependent LTD is Not Enhanced in DXKO Mice

To investigate whether excessive accumulation of drebrin E or the absence of drebrin A is involved in enhanced mGluR-dependent LTD in DAKO mice.

We have shown that LFS did not induce LTD in adult brain of either WT or DXKO mice (Figure 1F). This suggests that LFS-induced mGluR-dependent LTD in adult DAKO mice is not due to the absence of drebrin A but due to the excessive accumulation of drebrin E.

**Figure 6:**
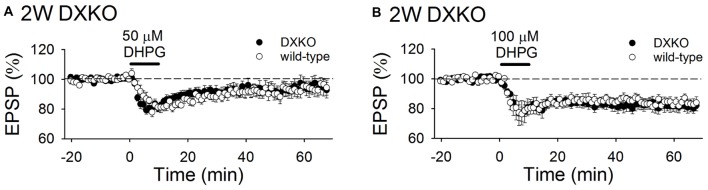
DHPG-induced LTD is not enhanced in developing DXKO mice. **(A)** Average time course of 50 μM DHPG-induced LTD in 2-week-old WT (*n* = 11 from four mice) and DXKO mice (*n* = 14 from six mice). **(B)** Average time course of 100 μM DHPG-induced LTD in 2-week-old WT (*n* = 14 from four mice) and DXKO mice (*n* = 9 from four mice).

Next, we examined the long-term effects of DHPG on synaptic transmission in developing DXKO mice. Changes in fEPSPs induced by 50 μM DHPG was not different between 2-week-old WT and DXKO mice (Figure 6A; WT, 94.9 ± 3.6%, *n* = 11 from four mice; DXKO, 95.6 ± 4.8%, *n* = 14 from six mice). LTD induced by 100 μM DHPG were not different between 2-week-old WT and DXKO mice (Figure 6B; WT, 84.7 ± 2.9%, *n* = 14 from four mice; DXKO, 82.0 ± 3.7%, *n* = 9 from four mice). This also suggests that excessive accumulation of drebrin E is responsible for the aberrant mGluR-dependent LTD observed in adult DAKO mice.

## Discussion

Here, we show that drebrin is required in NMDAR-dependent LTD induction and that isoform conversion from drebrin E to drebrin A is required to suppress group 1 mGluR-dependent LTD in the adult hippocampus. In the developing hippocampus, LFS induced NMDAR-dependent LTD in DAKO mice as well as WT mice. This indicates that isoform conversion of drebrin is not required for NMDAR-dependent LTD induction. On the other hand, LFS induced mGluR5-dependent LTD in DAKO mice throughout development and adulthood, but not in WT or DXKO mice. In DAKO mice, excessive accumulation of drebrin E was observed in synaptosomes in the developing and adult brain. Therefore, sustained expression of drebrin E in DAKO mice is responsible for induction of mGluR5-dependent LTD in the adult hippocampus.

LTD, an activity-dependent decrease in synaptic efficacy, is dominant in the developing brain and disappears in the adult brain (Dudek and Bear, 1993; Kemp et al., 2000). This suggests that LTD is important in developmental activity-dependent remodeling of neural connections that were generated according to genetic information. On the other hand, while LTD is classified into NMDAR-dependent LTD or mGluR-dependent LTD, in some brain disorders including anxiety disorder and depression, mGluR-dependent LTD is enhanced in adults (Niswender and Conn, 2010). Moreover, enhanced mGluR-dependent LTD is hypothesized to underlie mental retardation in fragile X syndrome, an autism spectrum disorder (Lüscher and Huber, 2010; Niswender and Conn, 2010; Bhakar et al., 2012). In addition, LTD becomes inducible again in aged animals that develop memory decline, suggesting that LTD might cause memory decline (Norris et al., 1996; Foster, 1999; Rosenzweig and Barnes, 2003). Thus, inappropriate expression of LTD could cause a decrease in brain function. In DAKO mice, enhanced mGluR5-dependent LTD is induced in adulthood. This is consistent with our previous studies showing that DAKO mice develop impairment of hippocampus-dependent contextual fear memory in adulthood (Kojima et al., 2016). In addition to the enhanced mGluR5-dependent LTD, LTP declines in adult DAKO mice (Kojima et al., 2016). The increase of mGluR5-dependent LTD and the decrease of LTP may both contribute to the memory impairment in adult DAKO mice. Collectively, the isoform conversion promotes normal adult brain function by suppressing mGluR5-dependent LTD.

Involvement of mGluR1 in mGluR-dependent LTD in the CA1 region is still in debate. Only mGluR5 is required to induce mGluR-dependent LTD (Fitzjohn et al., 1999; Eng et al., 2016), however, some articles reports that group 1 mGluR-dependent LTD requires mGluR1 activation (Volk et al., 2006; Kumar and Foster, 2007). The conditions in which mGluR1 is also required in mGluR-dependent LTD in the CA1 region have not been clearly understood. However, we found that YM 298198, a mGluR1 selective inhibitor, partially inhibited DHPG-induced LTD in developing DAKO mice (Figures 3C,D), suggesting that mGluR1 function is also enhanced in DAKO mice.

Long isoforms of Homer (Homer 1b, 1c, 2 and 3) are scaffold proteins for group 1 mGluRs, and they downregulate mGluR5 function in the normal brain (Ronesi et al., 2012). However, in fragile X model mice, Homer 1a, a short isoform that cannot multimerize with other Homer isoforms, binds to mGluR5 (Giuffrida et al., 2005) and disrupts interaction between mGluRs and long isoforms of Homer. This makes mGluRs constitutively active, resulting in the enhancement of mGluR5-dependent LTD (Ronesi et al., 2012). Thus, the induction of mGluR5-dependent LTD is strictly restricted in the normal brain. Both drebrin isoforms have two binding sites for Homers (Shiraishi-Yamaguchi et al., 2009), which bind to group 1 mGluRs and regulate their activity (Ronesi et al., 2012). This raised the possibility that drebrin, Homer and mGluRs form complexes. In addition, most drebrin A is bound to F-actin but a portion of drebrin E is not bound to F-actin in the adult brain (Aoki et al., 2005; Kojima et al., 2010). Therefore, excess drebrin E might bind to Homer-group 1 mGluR complexes in DAKO mice and modulate mGluR5 functions, resulting in the induction of abnormal group 1 mGluR-dependent LTD.

In developing WT and DAKO mice, LFS induced NMDAR-dependent LTD. However, LFS did not induce NMDAR-dependent LTD in P18–20 DXKO mice (Figure 1), suggesting that drebrin is necessary for NMDAR-dependent LTD in the hippocampus. Expression of drebrin E and NMDAR-dependent LTD is robust in the neonatal hippocampus and decreases in parallel with development (Dudek and Bear, 1993; Kemp et al., 2000; Aoki et al., 2005; Kojima et al., 2016); therefore, drebrin E might be more important for NMDAR-dependent LTD induction than drebrin A. However, drebrin E is not sufficient to induce NMDAR-dependent LTD, because NMDAR-dependent LTD is not induced in adult DAKO mice that express large amounts of drebrin E (Figures 4, 5).

Direct interactions between drebrin and intracellular signaling molecules critical for NMDAR-dependent LTD induction (e.g., calcineurin, protein phosphatase 1 and rap; Malenka and Bear, 2004) have not been elucidated. Previously, we reported that calcium influx through NMDARs is sufficient to induce NMDAR-dependent LTD in the P11–14 hippocampus and that calcium release through ryanodine receptors supports NMDAR-dependent LTD in the P18 and older hippocampus (Yasuda and Mukai, 2015). In addition, deletion of drebrin prevents extracellular calcium supply triggered by depletion of intracellular stores (Mercer et al., 2010). These raise a possibility that drebrin regulates calcium release from ryanodine receptors. Calcium might not be sufficiently supplied through ryanodine receptors in P18–20 DXKO mice, resulting in the failure of NMDAR-dependent LTD induction. In contrast, NMDAR-dependent LTD is induced in the hippocampus of P13–16 another line of drebrin KO mice (Willmes et al., 2017). We speculate that dependence on ryanodine receptors for the induction of NMDAR-dependent LTD becomes gradually higher during P15–17. However, calcium influx through NMDAR might still have been sufficient to induce LTD in P15–16 drebrin KO mice, presumably because LTD was induced by stimulation, which evokes fEPSPs of approximately 1.5 mV (Willmes et al., 2017) and NMDARs could have been fully activated during recordings, although we usually record fEPSPs with an amplitude of 0.3–0.4 mV.

Homer proteins bind to drebrin, and regulate calcium release from internal stores by binding to ryanodine receptors (Gasperini et al., 2009; Pouliquin and Dulhunty, 2009); therefore, involvement of drebrin-Homer complexes in calcium mobilization in dendritic spines warrants further investigation.

We have previously reported that spine length of the apical dendrite of CA1 pyramidal cells is significantly longer in adult DAKO mice than that in WT mice (Kojima et al., 2016). Since these mice exhibited abnormal LTD, as well as impaired LTP, there might be a correlation between LTP/LTD and spine length. Although the causal relationship is still unclear, fmr1-KO mice, an animal model of autism spectrum disorders, show abnormal spine configurations (elongated spines and increased density) and also show the enhanced mGluR-dependent LTD in the hippocampus. Thus, it is possible that the abnormal regulation of spine morphology leads to synaptic dysfunction in complex psychiatric disorders. Therefore, to determine which possibility is likely, DXKO mice should be useful. Changes in spine structures in DXKO mice should be investigated in the future.

## Data Availability

Datasets are available on request.

## Author Contributions

HYas, NK and TS conceived and designed the experiments. NK and KS generated DXKO mice. HYas, HYam, KH and NK performed the experiments and analyzed the data. All authors wrote the article.

## Conflict of Interest Statement

The authors declare that the research was conducted in the absence of any commercial or financial relationships that could be construed as a potential conflict of interest.
