# Supplementary material for: Drebrin Isoforms Critically Regulate NMDAR- and mGluR-Dependent LTD Induction
Source: Front Cell Neurosci. 2018 Oct 8;12:330. doi: 10.3389/fncel.2018.00330 (PMC6186840; doi:10.3389/fncel.2018.00330)
Supplement: Supplementary file 1 [file Presentation_1.pdf]

Supplementary Material

Drebrin isoforms critically regulate NMDAR- and mGluR-dependent LTD induction

Hiroki Yasuda, Nobuhiko Kojima, Kenji Hanmaura, Hiroyuki Yamazaki, Kenji Sakimura, and Tomoaki Shirao*

*** Correspondence:** Dr. Tomoaki Shirao: tshirao@gunma-u.ac.jp

Supplementary Figures

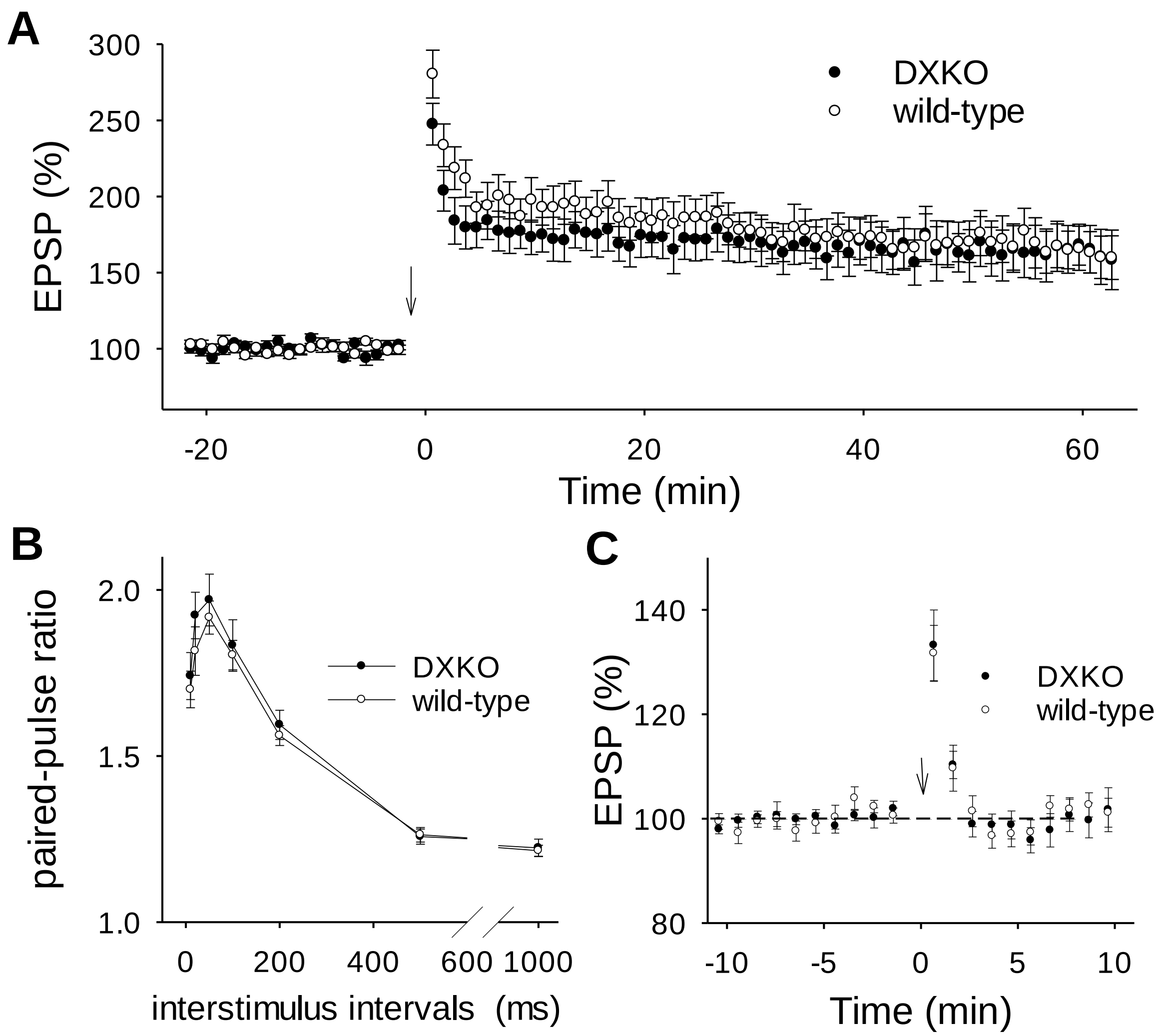

Supplementary Figure 1. LTP and presynaptic properties in developing DXKO mice.

(A) Summary of LTP experiment in P18–20 wild-type (n = 11 from four mice) and DXKO mice (n = 9 from four mice). LTP amplitude is similar between them. (B) Paired-pulse ratio was not different between wild-type and DXKO mice (wild-type, n = 10 from three mice; DXKO, n = 12 from three mice). (C) Posttetanic potentiation induced by a 100 Hz 1 sec stimulation was not different between wild-type and DXKO mice in the presence of 50 μM D-APV (wild-type, n = 9 from three mice; DXKO, n = 10 from three mice).

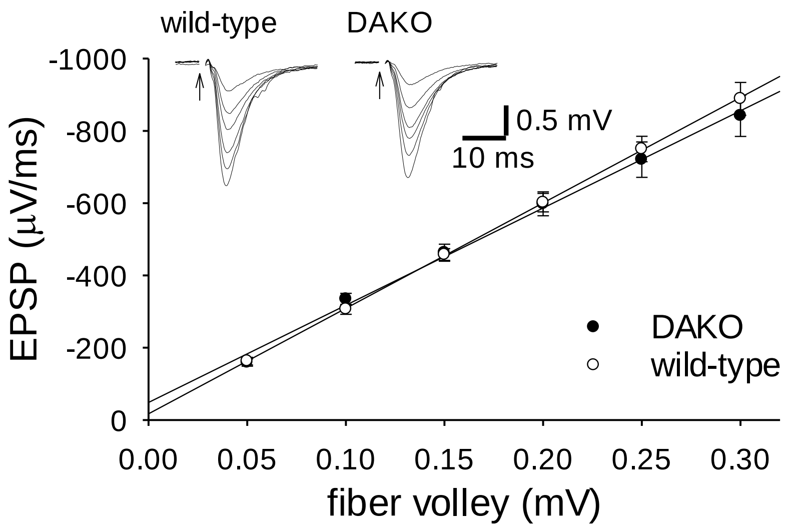

Supplementary Figure 2. Input-output relationship in developing wild-type and DAKO mice.

The input–output relationship is similar in postnatal 2-week-old wild-type and DAKO mice (wild-type, n = 11 from four mice; DAKO, n = 13 from four mice).

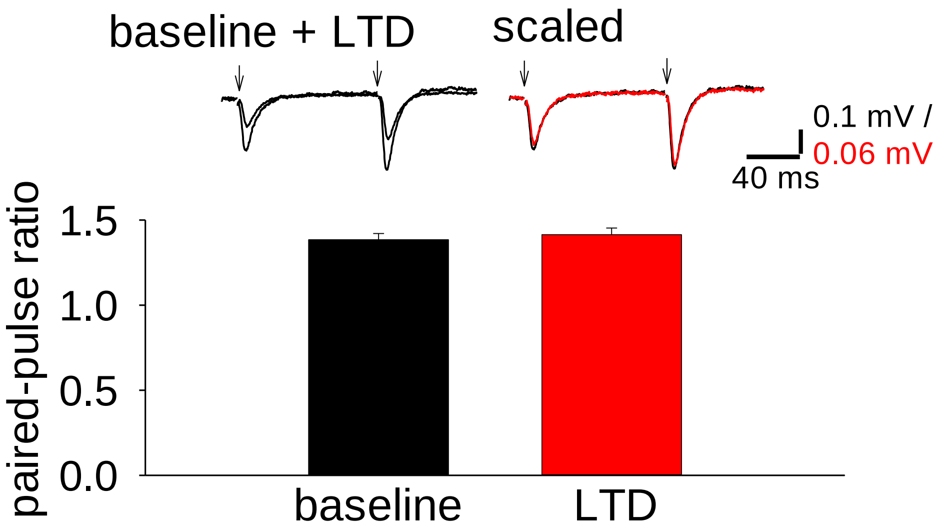

Supplementary Figure 3. LTD in adult DAKO mice is not associated with paired-pulse ratio.

Average of paired-pulse ratios before and 60 min after LFS in adult DAKO mice. No significant difference was detected in paired-pulse ratios before and 60 after LFS, suggesting that minimal presynaptic changes are involved in LTD in the adult hippocampus of DAKO mice (n = 5 from two mice).

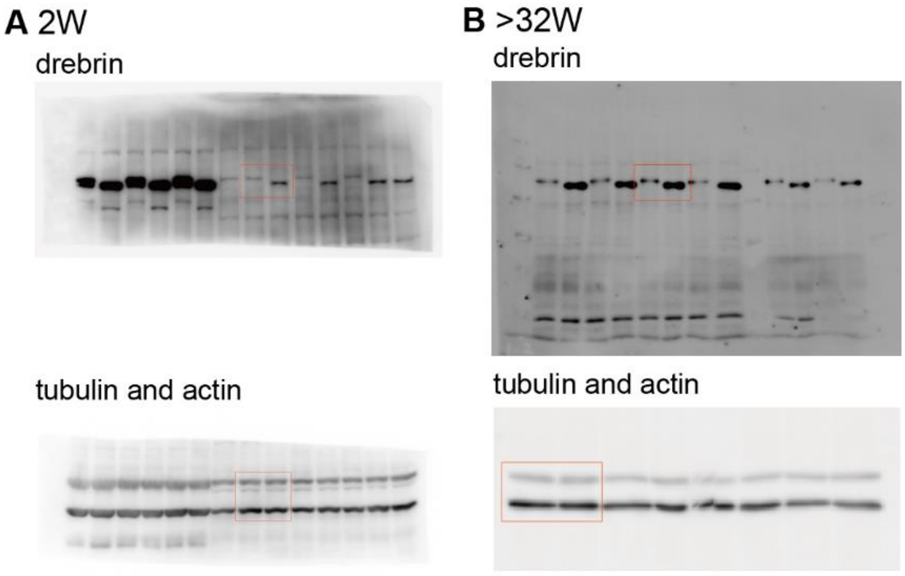

Supplementary Figure 4. Original images of western blot gels shown in Figures 5A (A) and 5B (B).
